# Around the Hospitals

**Published:** 1895-05-18

**Authors:** 


### AROUND THE HOSPITALS.

[NOTICE TO THE MANAGERS OF INSTITUTIONS.—Special space is now reserved for the insertion of notices of hospital meetings and festivals. The Editor requests that all notices may reach THE HOSPITAL Office, 428, Strand, at least one week before the date of each meeting.]

**The Perth City and County Infirmary.**—This hospital received 677 patients during the past year, and 756 were treated in the out-patients' department. We learn that 167 infectious cases were included amongst the patients, showing that the wisely-condemned system of receiving such cases in a general hospital still prevails at Perth. The fact that six nurses were set apart to nurse these cases shows that caution was exercised, but points to an unnecessarily large expenditure of resources, which would not occur under other arrangements. The ordinary income during the past year amounted to £2,475, and the ordinary expenditure to £3,530. The deficiency thus shown, and which will have to be met by encroaching on invested funds, we trust the subscribers will prevent in the future by increased liberality.

**The Devonshire Hospital and Buxton Bath Charity.**—The history of this institution for the treatment of rheumatic and kindred diseases is surrounded with interest. The hospital is the outcome of a charity believed to have existed as long ago as 1572 to assist poor visitors to Buxton with pecuniary aid. The Devonshire Hospital is now a large institution, with 300 beds. The buildings were originally stables, which, through the generosity of the Duke of Devonshire, to whom they belonged, were placed at the service of the trustees of the charity for conversion into a hospital. The design of the reconstructed and added-to structure is unique. The buildings surround a central space of half an acre, covered by an immense domed roof. This central space is used as a recreation ground, and forms a valuable addition to the resources of the hospital in cold or bad weather. A large number of patients come from the cotton manufacturing districts. The managers of the Cotton Districts' Fund have a prior right to send patients for treatment, as they are not only large contributors to the charity, but provided £24,000 towards the enlargement of the hospital. The average duration of a patient's visit is three weeks, during which time a marked improvement is usually visible. The treatment wisely includes an ample diet, many of the patients having suffered previous to their admission from insufficiency of food. The number of patients within the hospital at one time varies to a wonderful degree according to the season. During January only 53 patients may be within the walls, which contain 257 in August. This inequality in numbers must render the management a difficulty, which is further enhanced by the reception of a certain number of surgical cases, to whom the hospital extends most valuable and necessary aid. The financial condition of the hospital for the past year is satisfactory. The income has shown an improvement under all heads, and collectively has exceeded the expenditure by £487. The income amounted to £6,732 and the expenditure to £6,132, not including £113 transferred for investment. The number of in-patients amounted to 2,656, and the out-patients to 197. The cost per patient per week is estimated at 3s. 11d. The report of this most useful charity is most interesting reading and well drawn up, and, therefore, the fact that the accounts are not prepared after the uniform system is all the more to be regretted.

**The Grantham Hospital.**—We are glad to learn that during the past year improvements at the institution are visible in every direction. We should have been glad to have learnt the directions in which they have been effected, but information is very scantily given in the report, and the hospital cannot claim comparison with the best administered institutions until it adopts the uniform system of accounts, which alone render it possible to effect such comparison. We point out these deficiencies because we are aware that Grantham aspires to be in the van of hospital progress, and, therefore, is in a position when criticism is useful. The hospital has secured the services of an excellent matron; its expenses have been reduced, its income increased, and a cordial feeling towards it established in the neighbourhood. During the past year 236 patients have been treated in the wards—an increase of 37 on the numbers in 1893. Apparently, no out-patients are received. The ordinary income last year amounted to £1,544, and the ordinary expenditure to £1,330. A sum of £280 was spent on improvements and additions. It was necessary to realise £547 to pay off the debt to the treasurer, and meet extra expenses. It would be well if the report contained a statement of cost per patient and per bed in the hospital, particulars which the best drawn up reports contain.

